# Cell-free chromatin particles released from dying cells inflict mitochondrial damage and ROS production in living cells

**DOI:** 10.1038/s41420-023-01728-z

**Published:** 2024-01-15

**Authors:** Gorantla V. Raghuram, Bhabesh Kumar Tripathy, Kartikeya Avadhani, Snehal Shabrish, Naveen Kumar Khare, Relestina Lopes, Kavita Pal, Indraneel Mittra

**Affiliations:** 1 Translational Research Laboratory Advanced Centre for Treatment, Research and Education in Cancer, Tata Memorial Centre, Kharghar, Navi Mumbai, 410210 India; 2 Homi Bhabha National Institute, Anushakti Nagar, Mumbai, 400094 India

**Keywords:** Ageing, Circulation

## Abstract

Mitochondrial damage and the resultant oxidative stress are associated with neurodegenerative diseases, ageing, and cancer. However, the triggers of mitochondrial damage remain unclear. We previously reported that cell-free chromatin particles (cfChPs) released from the billions of cells that die in the body every day can readily enter healthy cells and damage their DNA. Here, we show that cfChPs isolated from the sera of healthy individuals, when applied to NIH3T3 mouse fibroblast cells, cause physical damage to mitochondrial DNA (mtDNA). cfChPs also induce ultrastructural changes, increase mitochondrial mass, alter mitochondrial shape, upregulate mitochondrial outer membrane protein translocase of the outer membrane 20, and change mitochondrial membrane potential. Furthermore, a marked increase was observed in mitochondrial superoxide (ROS) production, as detected by MitoSOX Red, and intracellular superoxide dismutase-1 activation. ROS production was also activated when a conditioned medium containing cfChPs released from hypoxia-induced dying NIH3T3 cells was applied to healthy NIH3T3 cells. ROS activation was significantly reduced when the conditioned medium was pre-treated with three different cfChP-deactivating agents: anti-histone antibody-complexed nanoparticles, DNase I, and the novel pro-oxidant combination of the nutraceuticals resveratrol and copper. Given that 1 × 10^9^–1 × 10^12^ cells die in the body every day, we hypothesise that cfChPs from dying cells are the major physiological triggers for mtDNA damage and ROS production. Deactivation of cfChPs may provide a novel therapeutic approach to retard ageing and associated degenerative conditions linked to oxidative stress.

## Introduction

The predominant source of intracellular ROS is believed to be the mitochondrial electron transport chain during oxidative phosphorylation [1]. In addition, external factors such as UV radiation, chemicals, heat, and pH stress can lead to the production of mitochondrial ROS [2–5]. Under physiological conditions, ROS levels are regulated by the balance between their generation and elimination by several antioxidant enzymes and non-enzymatic defence systems [6]. Oxidative stress can result from the presence of excess ROS or their inefficient elimination. One of the major triggers of excess ROS production is damage to mitochondria, particularly mtDNA [7, 8]. Excess ROS production can irreversibly damage mtDNA, proteins, and membrane lipids, resulting in mitochondrial dysfunction and ultimately cell death [9]. Chronic mitochondrial dysfunction is associated with ageing and several degenerative conditions including diabetes, cardiovascular diseases, neurodegenerative disorders, and cancer [10–14].

Several hundred billion-to-trillion cells die in the body daily [15, 16] and the cell-free chromatin particles (cfChPs) released from these cells enter extracellular compartments, including the circulation [17]. We previously reported that cfChPs can readily enter cells and damage their DNA [18, 19]. In this study, we provide evidence that cfChPs are a major trigger for ROS production because of their ability to readily enter healthy cells and inflict damage to mitochondria, especially mtDNA. We also provide evidence that mtDNA damage and ROS production can be minimised by treatment with cfChP-inactivating agents, suggesting therapeutic opportunities for multiple conditions associated with oxidative stress.

## Results

### cfChPs are readily internalised by NIH3T3 cells

**Fig. 1:**
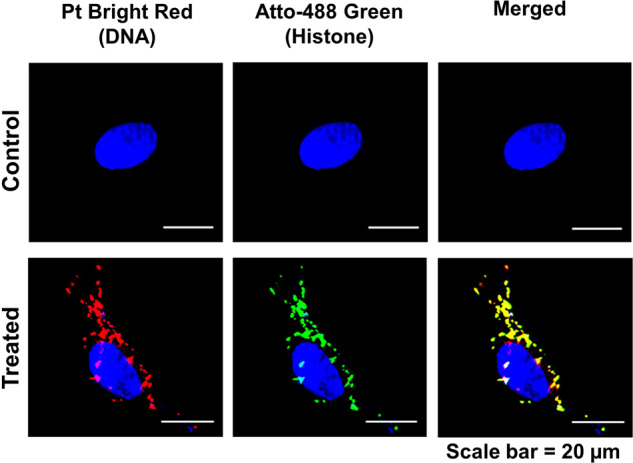
Rapid uptake of cfChPs by NIH3T3 cells at 4 h. NIH3T3 cells were treated with 10 ng cfChPs that were isolated from the sera of healthy individuals and dually labelled within the DNA (Platinum Bright 550) and the histones (ATTO-488). Images were acquired on a spectral bioimaging system (Applied Spectral Imaging). Dually labelled cfChPs are clearly seen in the cytoplasm. The particles appear relatively large, which is most likely due to linking-up of multiple cfChPs to form concatemers as previously described by our group [18].

cfChPs isolated from the sera of healthy individuals were fluorescently dually labelled in their DNA and histones with Platinum Bright 550 and ATTO-488, respectively, and added to cultured NIH3T3 cells (10 ng equivalent of DNA). Abundant fluorescent signals were detected in the cytoplasm of the treated cells at 4 h (Fig. 1). All experiments described below were conducted for 4 h. This experiment was performed multiple times.

### cfChPs inflict presumptive mtDNA damage: studies using whole cells

**Fig. 2:**
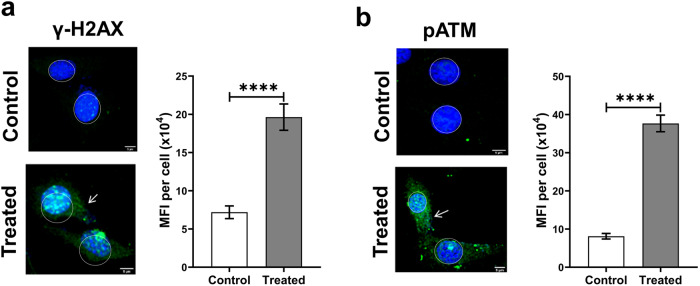
cfChP treatment of NIH3T3 cells induces presumptive mitochondrial DNA damage as observed at 4 h. **a** Representative images of untreated control and cfChP-treated (10 ng) cells stained with antibody against γH2AX (green). γH2AX expression is visibly increased in the cytoplasm of cfChP-treated cells (arrows). MFI was quantitatively analysed after gating and excluding nuclear fluorescence and the results reveal significantly higher MFI for γH2AX in treated cells as compared to untreated controls. Notably, cfChPs induced the expected activation of H2AX in the nuclei of treated cells, as previously reported by our group [18]**. b** Representative images of untreated control and cfChP-treated (10 ng) cells stained with antibody against pATM (green). Expression of pATM is visibly increased in the cytoplasm of cfChP-treated cells (arrows). MFI was quantitatively analysed after gating and excluding the nuclear fluorescence, which revealed significantly higher MFI for pATM in treated cells as compared to untreated controls. cfChPs induced the expected activation of ATM in the nuclei of the treated cells as previously reported by our group [18]. Experiments were repeated twice, and all images were acquired under the same settings. Results represent mean ± SEM values. Scale Bar - 5 µm. Data were analysed using Student’s *t*-tests. ***p < 0.001, ****p < 0.0001.

To investigate whether the internalised cfChPs induced mtDNA damage, NIH3T3 cells were treated with cfChPs (10 ng) and the treated cells were immune stained with antibodies against γH2AX and pATM. Numerous γH2AX and pATM fluorescence signals could be detected in the cytoplasm of treated cells in an amount significantly higher than those detected in untreated control cells (*p* < 0.0001; Fig. 2). The activation of H2AX and ATM in the cytoplasm, resulting from mtDNA damage, is described below.

### cfChPs inflict mtDNA damage: studies using isolated mitochondria

**Fig. 3:**
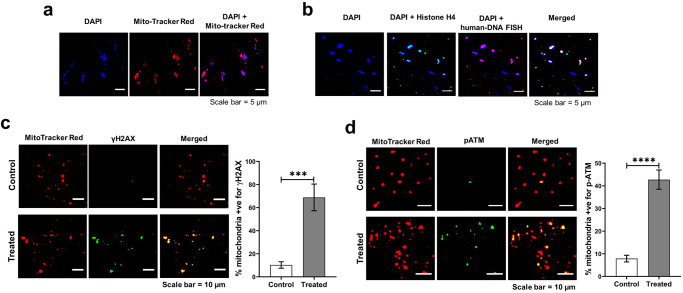
Mitochondria isolated from cfChP-treated cells show an intimate association with cfChPs. **a** Isolated mitochondria represented by MitoTracker Red co-localise with DAPI signals; **b** Immuno-FISH performed on isolated mitochondria using antibody against histone H4 and a human DNA FISH probe show co-localisation of cfChPs with mitochondria. **c** Mitochondria isolated from cfChP-treated cells show phosphorylated H2AX signals that co-localise with mitochondria, represented by MitoTracker Red. Few γH2AX signals are seen in untreated control cells. Histograms represent quantitative analysis of co-localising signals in control and treated cells. **d** Mitochondria isolated from cfChP-treated cells show phosphorylated ATM signals, which co-localise with mitochondria, represented by MitoTracker Red. Few pATM signals are seen in untreated control cells. Histograms represent quantitative analysis of co-localising signals in control and treated cells. Results are represented as mean ± SEM values and data were analysed using Student’s *t-*tests. ***p < 0.001; ****p < 0.0001.

The mitochondria were isolated from NIH3T3 cells that had been treated with cfChPs (10 ng) for 4 h. The isolated mitochondria were clearly identifiable as DAPI-positive particles which co-localised with MitoTracker Red (Fig. 3a). The isolated mitochondria were analysed by immunofluorescence in situ experiments using fluorescent antibodies against histone H4 and a whole genomic human DNA probe. Negative control experiments, not shown here, were performed to confirm that the DNA FISH probe did not cross-hybridise with mouse DNA. Strictly co-localised fluorescent signals of histone H4 and human DNA with DAPI indicated that cfChPs were in close contact with mouse mitochondria, as indicated by DAPI-positive signals (Fig. 3b). Dual staining of the isolated mitochondria using antibodies against γH2AX and MitoTracker Red and pATM and MitoTracker Red revealed strict co-localisation, indicating that cfChPs associated with mitochondria had inflicted mtDNA damage (Fig. 3c, d). When compared with isolated mitochondria from untreated NIH3T3 cells, the number of γH2AX and pATM signals in the mitochondria isolated from treated cells were significantly elevated (*p* < 0.001 and *p* < 0.0001, respectively).

### cfChPs damage other mitochondrial components

Next, we examined whether cfChPs induce damage to other mitochondrial components in addition to mtDNA.

#### Ultrastructural changes

**Fig. 4:**
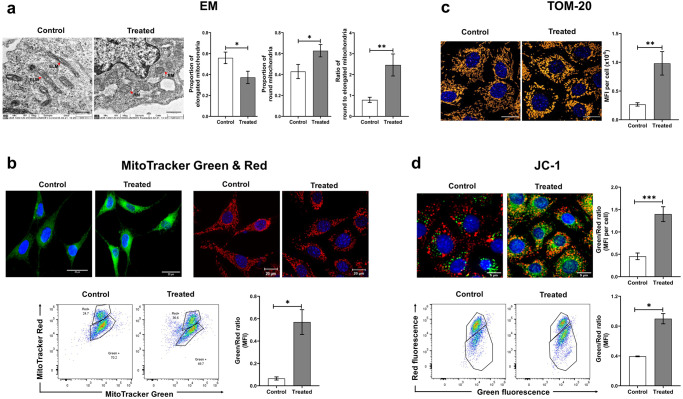
Various mitochondrial abnormalities are induced by cfChPs in treated cells as observed at 4 h. **a** Representative EM images of untreated and cfChP-treated (10 ng) cells reveal ultrastructural changes in the mitochondria. Control cells contain elongated mitochondria (red arrows), whereas cfChP-treated cells harbour mostly round-shaped mitochondria (red arrows; scale bar - 1 µm). ELM = Elongated Mitochondria, RM = Round Mitochondria. Histograms represent quantitative analysis of ultrastructural changes and the ratio of round-to-elongated mitochondria in treated and control cells. **b** Evaluation of mitochondrial dysfunction by immunofluorescence and flow cytometry using mitochondrial dyes. Representative microphotographs of mitochondrial mass (MitoTracker Green) and of mitochondrial dysfunction (MitoTracker red) in cfChP-treated (10 ng) and control cells. Quantitative analysis (MFI) of the ratio of mitochondrial mass and mitochondrial dysfunction by flow cytometry. The latter shows a significant increase in ratio of green-to-red fluorescence in cfChP-treated cells compared to untreated cells. **c** Representative confocal microphotographs of the mitochondrial outer membrane marker TOM20 in cfChP-treated (10 ng) and untreated cells. TOM20 expression is visibly higher in the cfChP-treated cells compared to untreated cells. (Scale bar - 20 µm, pseudo colour - orange). Quantitative analysis (MFI) of TOM20 expression in control versus cfChP-treated cells reveals a significant increase in the MFI in treated cells, indicating loss of mitochondrial membrane integrity as compared to control cells. **d** Representative confocal microphotographs of mitochondrial membrane potential marker JC-1 in cfChP-treated (10 ng) and untreated cells. Quantitative analysis (MFI) shows increased green-to-red fluorescence ratio, signifying depolarised mitochondrial membranes. Quantitative MFI analysis by flow cytometry reveals a higher green-to-red fluorescence ratio in cfChP-treated cells compared to control cells. All the experiments were repeated twice. Results are represented as mean ± SEM values and data were analysed using Student’s *t-*tests. * = *p* < 0.05, ** = *p* < 0.01, *** = *p* < 0.005.

When examined under transmission electron microscopy, untreated control cells harboured mitochondria that were normal and elongated in shape. In contrast, the mitochondria of cfChP-treated cells were mostly round, signifying mitochondrial damage [20]. In Fig. 4a, the proportions of elongated and round mitochondria in control and treated cells are depicted as histograms. Quantitative estimation of the ratio of round-to-elongated mitochondria showed a three-fold increase in favour of round mitochondria, indicating increased mitochondrial damage in the treated cells (Fig. 4a).

#### Increase in mitochondrial mass and mitochondrial shape

Cells upregulate mitochondrial biogenesis in response to mitochondrial damage in the form of increased mitochondrial mass [21]. MitoTracker Green has been widely used to assess mitochondrial mass, as it stains mitochondria irrespective of their membrane potential [22]. A marked increase in MitoTracker Green fluorescence was observed in cells treated with cfChPs, indicating that mitochondrial mass increased as a result of the damage inflicted by cfChP treatment (Fig. 4b). MitoTracker Red can detect changes in mitochondrial shape that correlate with mitochondrial damage [20, 23]. Confocal microscopy of cfChP-treated cells stained with MitoTracker Red revealed diffuse fluorescence signals representing mitochondrial fragmentation [20]. In contrast, untreated control cells exhibited a distinct mitochondrial network (Fig. 4b). Flow cytometry analysis of cells dually labelled with MitoTracker Green and Red was performed to determine the relative proportions of functional (MitoTracker Green^high^ and MitoTracker Red^high^) and dysfunctional (MitoTracker Green^high^ and MitoTracker Red^low^) mitochondria in response to treatment with cfChPs. Quantitative analysis revealed an increased ratio of MitoTracker Green^high^ and MitoTracker Red^low^ populations in cfChP-treated cells compared to that in untreated cells. These findings provide additional evidence of mitochondrial dysfunction (Fig. 4b).

#### Upregulation of mitochondrial outer membrane protein TOM20

TOM20 acts as an import receptor that belongs to the family of TOM proteins and allows movement of proteins across the mitochondrial outer membrane into the inner membrane space [24]. TOM20 upregulation accompanied with increased biogenesis and excessive import of proteins is a sign of dysfunctional mitochondria [25]. We observed a significant increase in TOM20 expression in cfChP-treated cells compared to that in the controls by confocal microscopy (Fig. 4c). Quantitative analysis, expressed as mean fluorescence intensity (MFI) per cell, showed a three-fold increase in TOM20 expression in cfChP-treated cells compared to that in untreated control cells (*p* < 0.01; Fig. 4c).

#### Altered mitochondrial membrane potential

Low mitochondrial membrane potential is associated with mitochondrial damage [26]. Mitochondrial depolarisation was investigated as an indicator of mitochondrial damage using JC-1 (5, 5′, 6, 6′-tetrachloro-1, 1′, 3, 3′-tetraethyl benzimidazolylcarbocyanine iodide), a mitochondrial membrane fluorescent dye. JC-1 aggregates in healthy mitochondria with high membrane potential and emits red fluorescence. On the other hand, in dysfunctional mitochondria, JC-1 appears as a monomer and emits green fluorescence since it has a relatively low membrane potential [27]. The representative images in Fig. 4d show that while control cells emitted predominantly red signals, cfChP-treated cells emitted significant green signals in addition to red signals. Quantitative analysis revealed a highly significant increase in the ratio of green-to-red fluorescence signals in cells treated with cfChPs (*p* < 0.001; Fig. 4d). These results were validated using flow cytometry, which revealed a two-fold increase in the ratio of green-to-red fluorescence in cfChP-treated cells compared to that in untreated cells (*p* < 0.05). Taken together, these results indicate that cfChPs induce mitochondrial damage in treated cells, leading to decreased mitochondrial membrane potential.

### cfChPs induce ROS production from mitochondria

**Fig. 5:**
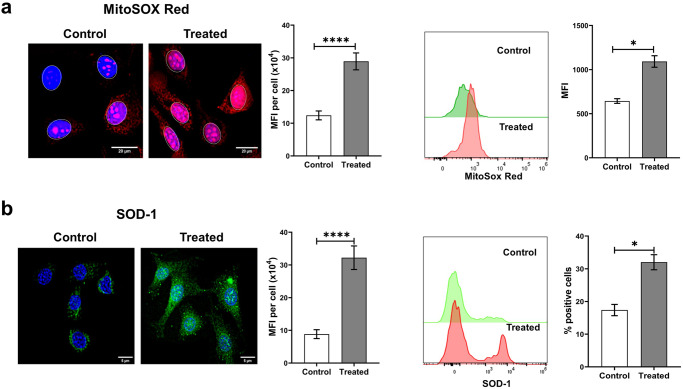
Treatment of NIH3T3 cells with cfChPs induces oxidative stress as detected at 4 h. **a** Representative fluorescence microscopy images of control and cfChP-treated (10 ng) cells stained with MitoSOX Red shows significant activation of ROS production in treated cells compared to untreated cells (scale bar = 5 µm). Results of the quantitative analysis of MFI (after gating the nuclei to exclude nuclear fluorescence) are shown as histograms. Flow cytometric analysis of MitoSox Red confirms a significant increase in treated cells compared to control cells. **b** Representative fluorescence microscopy images of control and cfChP-treated (10 ng) cells stained with superoxide dismutase-1 (SOD-1) antibody shows significant increase in the activation of SOD-1 in treated cells compared to untreated cells (scale bar = 5 µm). Results of the quantitative analysis (MFI) are shown as histograms. Flow cytometric analysis of SOD-1 confirms a significant increase in SOD-1 levels in treated cells compared to control cells. All the experiments were repeated twice. Results are represented as mean ± SEM values and data were analysed using Student’s *t-*tests. * = *p* < 0.05, *** = *p* < 0.005.

Mitochondrial damage, particularly mtDNA damage, is a major cause of excess ROS production [7, 8]. One of the primary forms of ROS generated in mitochondria are superoxide radicals [9] that can be readily detected by MitoSOX Red, a triphenylphosphonium (TPP + )-linked DHE compound that is selectively oxidised by superoxide radicals leading to the emission of red fluorescence [28]. To investigate whether mitochondrial damage, including mtDNA damage, led to increased ROS production, NIH3T3 cells were treated with cfChPs (10 ng) for 4 h followed by staining with MitoSOX Red. A significant increase in MitoSOX fluorescence was detected by microscopy (*p* < 0.0001; Fig. 5a). These results were validated by flow cytometry results, which revealed a two-fold increase in the MFI of MitoSox Red in cfChP-treated cells compared to that in untreated cells (*p* < 0.05). These data clearly indicate that cfChPs can induce increased ROS production resulting from damaged mitochondria and especially in response to mtDNA.

### cfChPs activate the antioxidant enzyme superoxide dismutase

Next, we performed immunofluorescence and flow cytometry assays to elucidate whether excess ROS production following the treatment of NIH3T3 cells with cfChPs led to an increase in levels of the antioxidant enzyme SOD-1. Fluorescence microscopy of cells treated with cfChPs (10 ng) for 4 h detected a highly significant increase in SOD-1 production (*p* < 0.0001; Fig. 5b). Flow cytometric analysis also showed increased SOD-1 expression in the treated cells (*p* < 0.05).

### Experiments using conditioned media from dying NIH3T3 cells

**Fig. 6:**
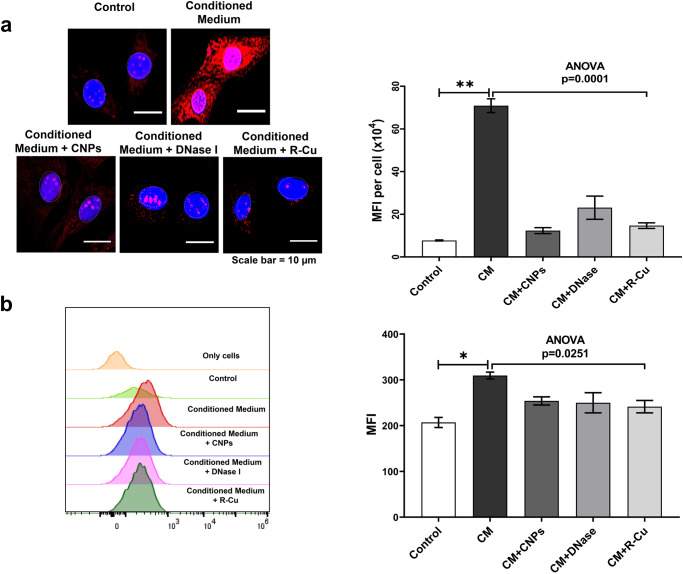
Activation of ROS production in NIH3T3 cells treated with conditioned medium containing cfChPs from hypoxia-induced-dying NIH3T3 cells in the presence and absence of cfChP inhibitors. **a** Representative fluorescence microscopy images showing activation of ROS production at 4 h, detected by MitoSox Red in NIH3T3 cells following treatment with conditioned medium (50 µl) from dying NIH3T3 cells (upper panel). ROS activation is inhibited by concurrent treatment with CNPs, DNase I, and R-Cu (lower panel). Results of the quantitative analysis (MFI) are shown as histograms. Since MitoSox Red generates the by-product mitoethidium, which intercalates within DNA and generates nuclear fluorescence, nuclei were gated and nuclear fluorescence was excluded from the MFI analysis. **b** Representative flow cytometry plots showing the activation of ROS production, represented by MitoSox Red, in NIH3T3 cells following treatment with conditioned medium (50 µl) from dying NIH3T3 cells. ROS activation is inhibited by concurrent treatment with CNPs, DNase I, and R-Cu. Results of quantitative analysis (MFI) are shown as histograms. Experiments were repeated twice. Results are represented as mean ± SEM values. In both **a** and **b** control and CM values were compared using two-tailed Student *t*-tests * < 0.05, ** < 0.01, *** < 0.001, **** < 0.0001. Statistical comparison between CM values and those of the three cfChP-deactivating agents was performed through a one-way analysis of variance (ANOVA). * < 0.05, ** < 0.01.

Based on our earlier finding that cfChPs spontaneously released from dying cells can be readily internalised by healthy cells [19], we treated NIH3T3 cells with conditioned medium containing cfChPs released from hypoxia-induced-dying NIH3T3 cells. The method for generating the cfChP-containing conditioned medium is described in the “Materials and Methods” section. Supplementary Fig 1 shows that cfChPs released from dually fluorescently labelled dying NIH3T3 cells were readily internalised by healthy NIH3T3 cells. First, we performed a dose-response experiment wherein we added increasing volumes of cfChP-containing conditioned medium to NIH3T3 cells, followed by MitoSOX Red staining to determine ROS production. Since MitoSox Red generates the by-product mitoethidium, which intercalates into DNA and generates nuclear fluorescence, nuclei were gated and nuclear fluorescence was excluded from the MFI analysis. Optimum ROS production was observed with 50 µl conditioned medium (Supplementary Fig. 2) and all further experiments were performed using this volume. To confirm that cfChPs in the conditioned medium were indeed responsible for ROS production, conditioned media were pre-treated with three different cfChP-deactivating agents: CNPs, DNase I, and R-Cu. As noted earlier, the marked activation of ROS in NIH3T3 cells following treatment with 50 µl of conditioned medium was abrogated by pre-treatment of the conditioned medium with all three cfChP-inactivating agents (*p* < 0.0001; Fig. 6a). Flow cytometry analysis also revealed downregulation of ROS production following pre-treatment with cfChP inhibitors (Fig. 6b). Notably, the degree of reduction detected by flow cytometry was somewhat lower than that detected by fluorescence microscopy. This is because nuclear fluorescence cannot be gated and is excluded from flow cytometry analysis.

## Discussion

Several hundred billion-to-trillion cells die in the body every day [15, 16] and cfChPs released from dying cells enter the extracellular compartments, including the circulation [17]. We previously reported that circulating cfChPs, or those released locally from dying cells, can readily enter healthy cells where they induce dsDNA breaks, inflammation, and apoptotic responses [18, 19]. The latter occurs via the mitochondria-mediated apoptotic pathway, as indicated by the increased levels of JC-1, cytochrome C oxidase, and caspase-3 [18].

The present study was based on the hypothesis that, in addition to DNA damage, internalised cfChPs would also damage the mitochondria. Indeed, treatment of NIH3T3 mouse fibroblast cells with cfChPs isolated from healthy human individuals led to the activation of H2AX and ATM signals that co-localised with those of mitochondria, verified by MitoTracker Red staining. This was further confirmed by experiments using mitochondria isolated from cfChP-treated NIH3T3 cells, wherein mtDNA damage was caused by the physical association of cfChPs with mtDNA. We speculate that the physical association of cfChPs with mitochondria is a consequence of a chemical interaction between the positively charged histones of cfChPs and negatively charged mtDNA. The binding of cfChPs to mitochondria leads to multiple signs of mitochondrial damage, including ultrastructural changes and changes in mitochondrial mass, shape, and function. These events lead to the activation of ROS production and the consequent upregulation of the antioxidant enzyme SOD-1. We propose that excess ROS production sets in motion a vicious cycle of increased mtDNA damage and ROS production, leading to oxidative stress that is associated with several pathological conditions such as neurodegenerative diseases, ageing, and cancer [7, 29, 30]. The mechanism of cfChP-induced mitochondrial damage and ROS production is illustrated in Supplementary Fig. 3.

We further demonstrated that cfChP-induced ROS production could be greatly reduced following pre-treatment with three different cfChP-inactivating agents. Of these three agents, a combination of the widely used nutraceuticals resveratrol and copper holds therapeutic promise. We have shown that combining small quantities of R and Cu leads to the production of oxygen radicals capable of deactivating cfChPs with multiple therapeutic effects [31–38].

Oxidative stress, resulting from increased ROS production, is the underlying cause of many severe disorders [10–14]. However, the cause of excess ROS production remains elusive. In this study, we showed that mitochondrial damage is induced by cfChPs, a type of extraneous agents. Given that 1 × 10^9^–1 × 10^12^ cells die in the body every day, our findings suggest that cfChPs from dying cells are the major physiological triggers for mtDNA damage and ROS production. Deactivation of cfChPs may provide a novel therapeutic approach to retard ageing and associated degenerative conditions linked to oxidative stress. In this context, we recently reported that the prolonged administration of resveratrol and copper can delay multiple biological hallmarks of ageing and neurodegeneration in C57Bl/6 mice [38].

## Materials and methods

### Reagents and antibodies

The commercial sources and catalogue numbers of the reagents and antibodies used in this study are listed in Supplementary Table 1.

### Isolation of cfChPs from the sera of healthy donors

#### Ethics approval

Institutional Ethics Committee (IEC) approval was obtained to collect blood samples from healthy volunteers, and signed informed consent was obtained using consent forms approved by the IEC (approval no. 900520).

#### Details of healthy donors

Ten millilitres blood sample was collected from five healthy donors and serum was separated. To reduce inter-sample variability, sera from five healthy volunteers were pooled to isolate cfChPs. Three volunteers were male (ages 26, 30, 35) and two were female (ages 28, 34).

#### Isolation of cfChPs

The cfChPs from sera of healthy individuals were isolated according to a protocol previously reported by our group [18]. When examined under an electron microscope, the isolated cfChPs were found to reveal a beads-on-a-string appearance typical of chromatin [18]. The amount of DNA in the cfChPs was estimated using the PicoGreen quantification assay (Thermo Fisher Scientific, Waltham, MA, USA), and cfChP concentrations were expressed in terms of their DNA content.

### Fluorescent dual labelling of cfChPs

The cfChPs were fluorescently dually labelled within their DNA using Platinum Bright 550 (red) and within their histone H4 with ATTO-488 (green) according to a protocol previously described by our group [18].

### Cell culture

We used NIH3T3 mouse fibroblast cells for this study. These cells were chosen because they have been widely used in biological research and our group has published multiple papers using this cell line [18, 19, 31] Cells were obtained from the American Type Culture Collection (ATCC, Manassas, VA, USA) and grown in Dulbecco’s Modified Eagle’s medium (DMEM; Gibco, Thermo Fisher Scientific, Catalogue No. 12800-017) containing 10% bovine calf serum (HyClone; Cytiva, Marlborough, MA, USA, Catalogue No. SH30073) and maintained at 37 °C in an incubated supplied with 5% CO_2_. For microscopy experiments, 1 × 10^5^ cells were seeded on coverslips in 1.5 ml DMEM; for flow cytometry, 2 × 10^5^ cells were seeded in 1.5 ml DMEM and incubated overnight before conducting the experiments.

### Fluorescence and confocal microscopy

For fluorescence microscopy, cells were imaged using a spectral bioimaging system (Applied Spectral Imaging, Carlsbad, CA, USA). Images were captured using a 40× air objective. All images were captured at the same exposure time to ensure uniform fluorescence intensity for comparison. Confocal images were acquired using a 63× oil objective on a Zeiss LSM 780 laser scanning microscope (Carl Zeiss, Oberkochen, Germany) or Leica SP8 confocal imaging system (Leica Microsystems, Wetzlar, Germany). The mean fluorescence intensity (MFI) of the images was measured using ImageJ (Rasband, W.S., U.S. National Institutes of Health, Bethesda, MD, USA). MFI values are represented in arbitrary units.

### Flow cytometry

For flow cytometry, cells were analysed on an Attune NxT flow cytometer (Thermo Fisher Scientific) using FlowJo*™* Software version 10.6 (BD, Ashland, OR, USA). NIH3T3 cells were gated using forward- and side-scatter parameters. At least 20,000 NIH3T3 cells were acquired and the threshold between negative and positive expression was defined using the fluorescence minus one (FMO) method. The median fluorescence intensity (MFI) of the respective dyes was then compared by histogram analysis of the untreated and treated cells.

### Detection of presumptive mtDNA damage in whole cells

Cells were stained with antibodies against γ-H2AX and pATM (Supplementary Table 1), counterstained with Vectashield DAPI, and mounted on slides. The images were acquired using a spectral bioimaging system (Applied Spectral Imaging). Mean fluorescence intensity (MFI) in the cytoplasm was measured after gating out the nuclear fluorescence. This experiment was performed twice.

### Detection of mtDNA damage in isolated mitochondria

#### Isolation of mitochondria

NIH3T3 cells were treated with 10 ng cfChPs for 4 h, and mitochondria were isolated from the treated cells using a Mitochondria Isolation Kit (Thermo Fisher Scientific; Supplementary Table 1) according to the manufacturer’s instructions. Mitochondria were also isolated from untreated control cells.

#### MitoTracker Red CMXRos staining of isolated mitochondria

Isolated mitochondria were cytospun onto a slide and fixed in a 2% paraformaldehyde (PFA) / 1.5% glutaraldehyde (GA) fixative mixture for 15 min on ice, followed by neutralisation in 0.3 M glycine buffer for 30 min at room temperature (~25 °C). The fixed smears were stained with 100 nM MitoTracker Red CMXRos for 15 min at room temperature and stained with DAPI (1 µg/ml) in Hanks Balanced Salt Solution (HBSS). Images were acquired using a spectral bioimaging system (Applied Spectral Imaging).

#### Immuno-FISH staining of isolated mitochondria to detect histones H4 and DNA

Isolated mitochondria were fixed as described above and processed for immunostaining using anti-histone H4 IgG and the appropriate secondary antibodies (Supplementary Table 1). The immunostained smears were fixed with a 2% PFA-1.5% GA mixture for 5 min on ice and processed for FISH using a custom-synthesised human DNA probe (Supplementary Table 1). Briefly, immune-stained slides were hybridised overnight with a human DNA probe at 37 °C and washed once with 0.4X sodium saline citrate (SSC) at 72 °C (±2 °C) for 2 min and 4X SSC in Tween-20 twice at room temperature for 5 min each. The slides were mounted with DAPI (1 µg/ml) in HBSS, and images were acquired using a spectral bioimaging system (Applied Spectral Imaging).

### Assessment of mitochondrial integrity by electron microscopy

Exponentially growing NIH3T3 cells (4 × 10^6^) were treated with cfChPs (10 ng) for 4 h. Control and treated cells were fixed with 3% glutaraldehyde in 0.1 M sodium cacodylate buffer (pH 7.2) for 2 h at 4 °C followed by 1% osmium tetroxide in 0.1 M sodium cacodylate buffer for 1 h at 4 °C. Samples were then dehydrated using alcohol at 4 °C and embedded in Araldite resin to prepare ultrathin sections that were mounted onto EM grids for imaging. The grids were examined under a JEM1400-Plus transmission electron microscope (JEOL, Tokyo, Japan). This experiment was performed twice.

### Assessment of mitochondrial mass using MitoTracker Green FM

#### Fluorescence microscopy

Following treatment with 10 ng cfChPs for 4 h, NIH3T3 cells were stained with 100 nM MitoTracker Green FM diluted in 2 ml HBSS (pH 7.4) for 15 min at 37 °C. The cells were washed with HBSS, counterstained with Hoechst, mounted on glass slides, and imaged using the spectral bioimaging system. This experiment was performed twice.

#### Flow cytometry

Cells were stained with 20 nM MitoTracker Green FM diluted in 2 ml HBSS (pH 7.4) for 15 min at 37 °C. The cells were washed twice with prewarmed HBSS and collected in FACS tubes using a cell scraper. Samples were analysed using an Attune NxT flow cytometer (Thermo Fisher Scientific).

### Assessment of mitochondrial shape using MitoTracker Red CMXRos

#### Fluorescence microscopy

Following treatment with cfChPs (10 ng) for 4 h, NIH3T3 cells were stained with MitoTracker Red CMXRos (100 nM) diluted in 2 ml of HBSS (pH 7.4) for 15 min at 37 °C. The cells were then washed thrice with PBS, fixed using 4% PFA, counterstained with Vectashield DAPI, and mounted onto glass slides. Images were acquired using an LSM 780 confocal microscope (Carl Zeiss). This experiment was performed twice.

#### Flow cytometry

Cells were stained with MitoTracker Red CMXRos (100 nM) diluted in 2 ml of HBSS (pH 7.4) for 15 min at 37 °C. The cells were washed twice with prewarmed HBSS and collected in FACS tubes using a cell scraper. Samples were analysed using an Attune NxT flow cytometer (Thermo Fisher Scientific). This experiment was performed once.

### Assessment of mitochondrial membrane protein translocase of the outer membrane 20 (TOM20)

NIH3T3 cells were treated with 10 ng cfChPs for 4 h and immunostaining for TOM20 was performed by fixing the cells with 4% PFA for 15 min at 37 °C followed by permeabilisation with 0.2% Triton X-100 for 30 min and blocking with 3% BSA for 1 h. Cells were immune-stained using anti-rabbit TOM20 primary antibody and appropriate secondary anti-rabbit FITC-conjugated antibody followed by mounting with Vectashield DAPI. Images were acquired using the Leica SP8 confocal imaging system (Leica Microsystems). This experiment was performed twice.

### Assessing mitochondrial membrane potential using JC-1 dye

#### Fluorescence microscopy

After treatment with 10 ng cfChPs for 4 h, NIH3T3 cells were stained with JC-1 dye in HBSS and incubated at 37 °C for 15 min. The cells were washed twice with prewarmed HBSS. Cells were counterstained with Hoechst, mounted on glass slides, and cell-associated fluorescence was detected using a spectral bioimaging system (Applied Spectral Imaging). This experiment was performed twice.

#### Flow cytometry

Cells were stained with JC-1 dye in HBSS and incubated at 37 °C for 15 min. The cells were washed twice with prewarmed HBSS and collected in FACS tubes using a cell scraper. Samples were analysed using an Attune NxT flow cytometer (Thermo Fisher Scientific). This experiment was performed once.

### Assessment of mitochondrial ROS production using MitoSOX Red

#### Fluorescence microscopy

NIH3T3 cells were treated with 10 ng cfChPs for 4 h and stained with 0.5 µM MitoSOX Red. Cells were placed in 2 ml HBSS for 15 min at 37 °C and washed with warm buffer. Cells were counterstained with Hoechst, mounted on glass slides, and cell-associated fluorescence was measured using a spectral bioimaging system (Applied Spectral Imaging). This experiment was performed twice.

#### Flow cytometry

Cells were stained with 0.5 µM MitoSOX Red diluted in 2 ml HBSS for 15 min at 37 °C, washed with pre-warm HBSS, and then collected in FACS tubes using a cell scraper. Samples were analysed using an Attune NxT flow cytometer (Thermo Fisher Scientific). This experiment was performed once.

### Assessment of superoxide dismutase (SOD-1) expression

#### Fluorescence microscopy

cfChP-treated NIH3T3 cells were fixed with 4% PFA for 15 min at 37 °C and then immune-stained using primary antibody against SOD-1 and appropriate secondary antibody according to the protocol described above. Cells were mounted with Vectashield DAPI and images were acquired using a spectral bioimaging system (Applied Spectral Imaging). This experiment was performed twice.

#### Flow cytometry

Cells were fixed with 4% PFA for 15 min at 37 °C and then stained using primary antibody against SOD-1 and an appropriate secondary antibody. Cells were analysed using an Attune NxT flow cytometer (Thermo Fisher Scientific). This experiment was performed once.

### Experiments using cfChPs released from hypoxia-induced-dying NIH3T3 cells

#### Procedure for collecting conditioned media from hypoxia-induced-dying NIH3T3 cells

A dual-chamber system was used to generate a culture medium containing cfChPs released from dying cells. Briefly, 2 × 10^5^ NIH3T3 cells were seeded on ThinCert^®^ cell culture inserts (pore size 400 nm; Greiner Bio-One, Kremsmünster, Austria) containing 1.5 ml DMEM, and were placed in a six-well culture plate. Cells were incubated at 37 °C for 24 h. The six-well plate with Thincert^®^ inserts was then transferred to a hypoxia chamber with 1% O_2_ to induce hypoxic cell death. After 48 h, the plate was removed from the hypoxia chamber and 700 μl DMEM was added to the lower chamber of the six-well plate below the ThinCert^®^ inserts. The plates were then incubated for 48 h at 37 °C in normoxic conditions to allow cfChPs <400 nm in size to seep into the medium in the lower chamber. The medium in the lower chamber was collected, divided into aliquots, and stored at −80 °C until further use.

#### Preparation of cfChPs deactivators

The following three cfChP deactivators were used: (1) pullulan-histone antibody nanoconjugates (CNPs): CNPs were synthesised according to our previous protocol [39] and used at a dose of 25 µg/1.5 ml; (2) DNase I, Bovine pancreatic DNase I (0.0005 U/ml) was procured from Sigma-Aldrich (St Louis, MO, USA; Catalogue No. DN25-1G); and (3) resveratrol-copper (R-Cu). Resveratrol (R) is a plant polyphenol with antioxidant properties [40], but acts as a pro-oxidant in the presence of copper (Cu) because of its ability to reduce Cu (II) to Cu (I), thereby generating free radicals [41, 42]. R-Cu can deactivate cfChPs in vitro by degrading their DNA component [19, 31]. We used R-Cu at a molar ratio of R (1 mM) and Cu (1 × 10^−7^ mM). Resveratrol (Sigma, St Louis, CA, USA; Catalogue No. R5010) solution (2 mM) was prepared in 30% ethanol (solution A): copper sulfate (MP Biomedicals, Illkirch, France; Catalogue No. 191415) solution (20 mM) was prepared in distilled water and serially diluted to 2 × 10^−8^ mM concentration (solution B). Solution A and solution B were mixed at a ratio of 1:10 to obtain a mixture containing R (1 mM) and Cu (1 × 10^−7^ mM). One hundred μl of this mixture was added to 1.5 ml of culture media.

#### Assessment of mitochondrial ROS production following treatment with conditioned medium

Aliquots were thawed for use in the dose/volume response experiments and ROS levels were measured by MitoSOX Red staining. A dose/volume of 50 µl was most effective in generating ROS (Supplementary Fig. 1). Subsequently, experiments were repeated following pre-treatment with three different cfChP deactivators: pullulan-histone antibody nanoconjugates (CNPs), DNase I, and a novel pro-oxidant combination of small quantities of resveratrol and copper (R-Cu; see above for details).

Conditioned media were individually pre-treated with the three cfChP deactivators for 1 h at 37 °C prior to addition to the NIH3T3 cells. ROS production in the NIH3T3 cells was measured after 4 h using MitoSox Red (as described above). This experiment was performed twice.

### Statistical analysis

All data are presented as mean ± standard error of the mean (SEM). Statistical analyses were performed using GraphPad Prism 8 software (GraphPad Software, Inc., La Jolla, CA, USA, Version 8.0). Data were compared using Student’s *t-*tests (two-tailed, unpaired) and one-way analysis of variance (ANOVA). Results with *p* < 0.05 were deemed statistically significant.

### Supplementary information

Supplementary Information

## Data Availability

All the data are included in the manuscript. Additional data will be provided upon request.
