# Supplementary material for: Cell-free chromatin particles released from dying cells inflict mitochondrial damage and ROS production in living cells
Source: Cell Death Discov. 2024 Jan 15;10:30. doi: 10.1038/s41420-023-01728-z (PMC10789803; doi:10.1038/s41420-023-01728-z)
Supplement: Supplementary file 1 — Supplementary Information [file 41420_2023_1728_MOESM1_ESM.pdf]

Supplementary Figures

Supplementary Fig. 1

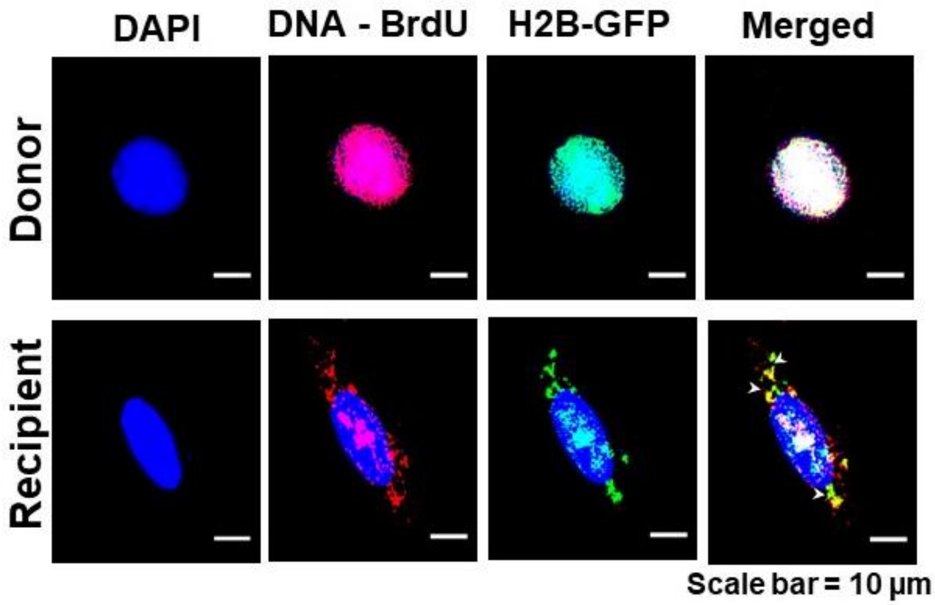

Legend to Fig S1. Representative fluorescence microscopy images showing bystander uptake of dually labelled cfChPs by NIH3T3 cells after 4 h. Donor NIH3T3 cells were dually labelled in their DNA with BrdU (red) and in their histones using CellLight Histone 2B-GFP (green). Healthy recipient NIH3T3 cells were incubated in a hypoxic chamber for 48 h and the conditioned medium (50 µl) containing dually labelled cfChPs was added.

Supplementary Fig. 2

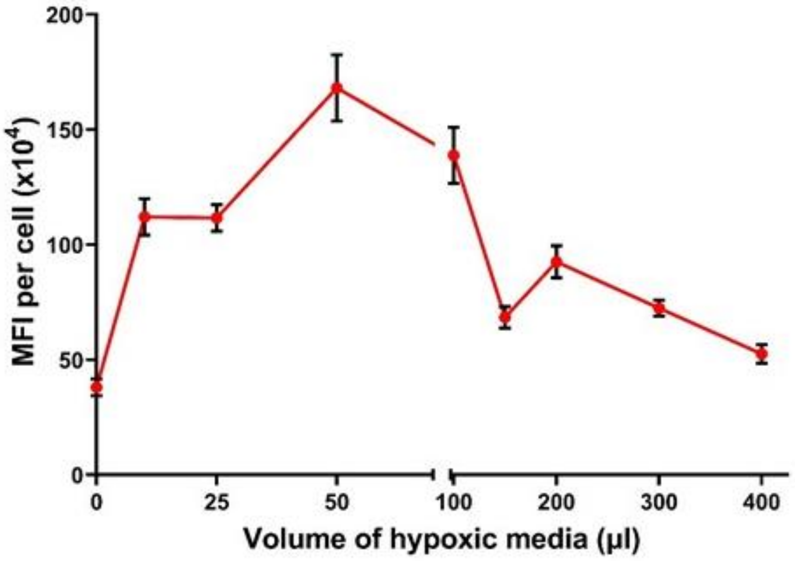

Legend to Fig S2. Dose/volume response analysis of ROS production as detected by MitoSOX at 4 h. NIH3T3 cells were treated with increasing volumes of conditioned medium containing cfChPs released from hypoxia-induced-dying NIH3T3 cells. Maximum activation of MitoSOX is seen in association with 50 µl of culture medium.

Supplementary Fig. 3

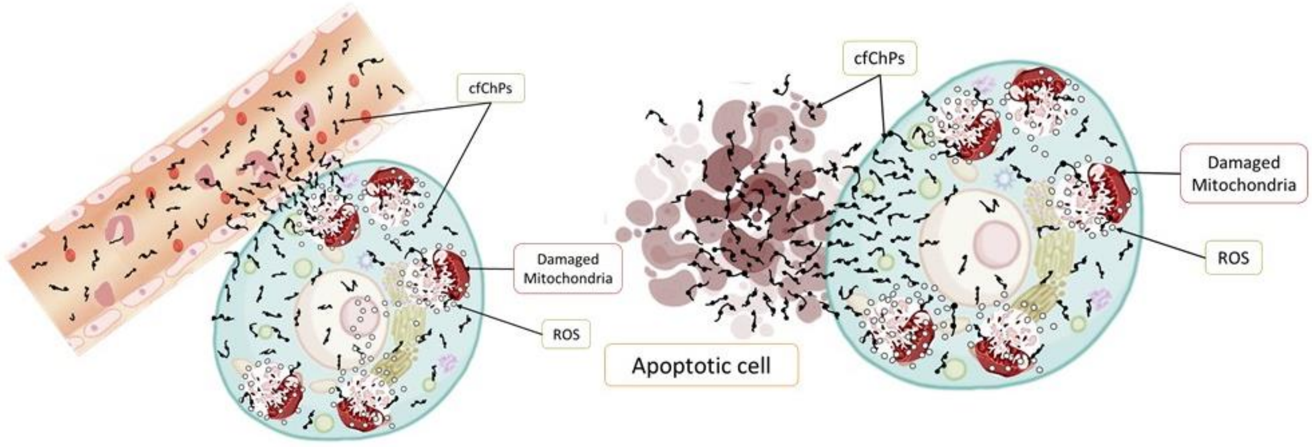

Legend to Fig S3. Illustration showing that cfChPs circulating in the blood (left-hand image) and those released locally from dying cells (right-hand image) can readily enter healthy cells to damage their mitochondria, leading to ROS production.

Supplementary Table 1.

Sources of reagents and antibodies used in this study.

List of reagents used.

Sl. No.	Reagent	Catalogue Number	Company
1	Platinum Bright 550 Red	GLK-004	Kreatech Diagnostics, Denmark
2	ATTO 488	AD 488	ATTO-TEC, GmbH, Germany
3	MitoSOX Red	M36008	Thermo Fisher Scientific, USA
4	MitoTracker Green FM	M7514	Thermo Fisher Scientific, USA
5	MitoTracker Red CMX Ros	M7512	Thermo Fisher Scientific, USA
6	Hoechst 33342	H21492	Thermo Fisher Scientific, USA
7	Vecta-Shield DAPI	101098-042	Vector Laboratories, USA
8	MitoScreen kit (JC-1)	551302	BD Biosciences, USA
9	Mitochondria Isolation Kit	89874	Thermo Fisher Scientific, USA

List of antibodies used

Sl. No.	Antibody	Catalogue Number	Company
1	Rabbit monoclonal anti- TOMM20 antibody	ab186735	Abcam, United Kingdom
2	Rabbit monoclonal anti-Superoxide	ab51254	Abcam, United Kingdom

	Dismutase-1 antibody		
3	Mouse monoclonal anti- γ-H2AX	05-636	Merck, GmbH, Germany
4	Mouse monoclonal anti-phospho-ATM (Ser 1981) antibody	05-740	Merck, GmbH, Germany
5	Goat Anti-Rabbit IgG H&L (FITC)	ab6717	Abcam, United Kingdom
7	Rabbit Anti-mouse (FITC)	AP160F	Merck, GmbH, Germany
